# Charting new paradigms for CAR-T cell therapy beyond current Achilles heels

**DOI:** 10.3389/fimmu.2024.1409021

**Published:** 2024-05-01

**Authors:** Ying Li, Zhenhua Hu, Yuanyuan Li, Xiaoyan Wu

**Affiliations:** ^1^ Department of Pediatrics, Union Hospital, Tongji Medical College, Huazhong University of Science and Technology, Wuhan, China; ^2^ Department of Health and Nursing, Nanfang College of Sun Yat-sen University, Guangzhou, China; ^3^ Zhongshan Institute for Drug Discovery, Shanghai Institute of Materia Medica, Chinese Academy of Sciences, Zhongshan, China; ^4^ Shanghai Institute of Materia Medica, Chinese Academy of Sciences, Shanghai, China; ^5^ University of Chinese Academy of Sciences, Beijing, China

**Keywords:** chimeric antigen receptor, immunotherapy, allogeneic universal CAR-T cells, *in vivo* CAR-T cell, Achilles heels

## Abstract

Chimeric antigen receptor-T (CAR-T) cell therapy has made remarkable strides in treating hematological malignancies. However, the widespread adoption of CAR-T cell therapy is hindered by several challenges. These include concerns about the long-term and complex manufacturing process, as well as efficacy factors such as tumor antigen escape, CAR-T cell exhaustion, and the immunosuppressive tumor microenvironment. Additionally, safety issues like the risk of secondary cancers post-treatment, on-target off-tumor toxicity, and immune effector responses triggered by CAR-T cells are significant considerations. To address these obstacles, researchers have explored various strategies, including allogeneic universal CAR-T cell development, infusion of non-activated quiescent T cells within a 24-hour period, and *in vivo* induction of CAR-T cells. This review comprehensively examines the clinical challenges of CAR-T cell therapy and outlines strategies to overcome them, aiming to chart pathways beyond its current Achilles heels.

## 1 Introduction

In recent years, chimeric antigen receptor-T (CAR-T) cell therapy has emerged as a pivotal immunotherapeutic approach, profoundly reshaping the treatment landscape of hematological malignancies. Engineered synthetic receptors, CAR-T cells empower T cells to selectively recognize and eliminate tumor cells independent of the major histocompatibility complex (MHC) (1, 2). Since the first FDA approval of a CAR-T cell product in 2017 (3), this therapy has witnessed rapid expansion in hematologic malignancies. As of now, six CAR-T cell products have received FDA approval, achieving remarkable complete remission (CR) rates of up to 80% in certain relapsed or refractory (R/R) B-cell malignancies (**Table 1**) (14, 15), while hundreds of CAR-T cell therapies are currently undergoing clinical trials (16, 17). Additionally, CAR-T cell therapy has shown notable success in treating autoimmune diseases, offering a 100% drug-free alternative to systemic lupus erythematosus in clinical trials (18, 19). The high remission rates and broad application across various diseases underscore the significance of CAR-T cell therapy as a pivotal tool in combating disease and reducing mortality rates (20, 21).

**Table 1:** FDA-approved CAR T-cell products.

Product	Target	CAR structure: Ag binding domain/Hinge/transmembrane/intracellular	Cost	Indication	Age	Approval	Pivotal trial	No. of Patients	Response	Toxicities (Grade≥3, %)	Ref
Kymriah (tisagenlecleucel)	CD19	Anti-CD19 scFv/CD8α/CD8α/4-1BB/CD3ζ	$475 000	R/RB-ALL	≤25y	Aug 30, 2017	ELIANA	75	ORR 81%, CR 60%	CRS:77 (46) NT:40 (13)	(4)
				R/R LBCL	Adult	May 1, 2018	JULIET	93	ORR 52%, CR 40%,	CRS:58 (22) NT:21 (12)	(5)
				R/R FL	Adult	May27, 2022	ElARA	97	ORR 86%, CR 69%	CRS:49 (0) NT:37 (3)	(6)
Yescarta (axicabtagene ciloleucel)	CD19	Anti-CD19 scFv/CD28/CD28/CD28/CD3ζ	$375 000	R/R LBCL	Adult	Oct 18, 2017	ZUMA-1	111	ORR 82%, CR 54%	CRS:93 (13) NT:64 (28)	(7)
				R/R FL	Adult	Mar 3, 2021	ZUMA-5	104	ORR 92%, CR 74%	CRS:82 (7) NT:59 (19)	(8)
Tecartus (brexucabtagene autoleucel)	CD19	Anti-CD19 scFv/CD28/CD28/CD28/CD3ζ	$373 000	R/R MCL	Adult	Jul 24, 2020	ZUMA-2	60	ORR 93%, CR 67%	CRS:91 (15) NT: 63 (31)	(9)
				R/R B-ALL	≥26y	Oct 1, 2021	ZUMA-3	71	ORR 71%, CR 56%	CRS:89 (24) NT:60 (25)	(10)
Breyanzi (lisocabtagene maraleucel)	CD19	Anti-CD19 scFv/IgG4/CD28/4-1BB/CD3ζ	$410 300	R/R LBCL	Adult	Feb 5, 2021	Transcend NHL001	256	ORR 73%, CR 53%	CRS:42 (2) NT:30 (10)	(11)
Abecma (idecabtagene vicleucel)	BCMA	Anti-BCMA scFv/CD8α/CD8α/4-1BB/CD3ζ	$419 500	R/R MM	Adult	Mar 26, 2021	KarMMa	128	ORR 73%, CR 33%	CRS:84 (5) NT:18 (3)	(12)
Carvykti (ciltacabtagene autoleucel)	BCMA	Anti-BCMA scFv/CD8α/CD8α/4-1BB/CD3ζ	$465 000	R/R MM	Adult	Feb 28, 2022	CARTITUDE-1	113	ORR 97%, CR 67%	CRS:95 (5) NT:21 (10)	(13)

R/R, refractory or relapsed; B-ALL, B-cell acute lymphoblastic leukemia; LBCL, large B Cell Lymphoma; FL, follicular lymphoma; MCL, Mantle-cell lymphoma; MM, multiple myeloma.

However, despite significant achievements, the widespread application of CAR-T cell therapy encounters numerous challenges. Firstly, the individualized customization and labor-intensive manufacturing process of CAR-T cells result in high costs and prolonged production cycles, limiting patient affordability and treatment accessibility (22–24). Moreover, efficacy concerns such as tumor antigen modulation, CAR-T cell persistence, and the immunosuppressive tumor microenvironment (TME) contribute to initial resistance or relapse in some patients (25–27). Additionally, safety issues including the risk of secondary cancers post-treatment, on-target off-tumor toxicity, and immune effector responses triggered by CAR-T cell activation further impede broad adoption (28–30). Specifically, the FDA requires the addition of warning information regarding the risk of secondary cancers post-treatment to the label of CAR-T cell products, introducing a new Achilles’ heel to CAR-T cell therapy.

To tackle these challenges, various strategies have been explored. These include constructing allogeneic CAR-T cells with enhanced potency and safety through leveraging the multiple gene editing functions of CRISPR-Cas9 and base editing (31, 32), *in vivo* induction of CAR-T cells by nanocarriers and optimized lentiviral vectors (33, 34), and the rapid production of potent CAR-T cells employing the FasTCAR platform or the MASTER scaffolds (35, 36). These efforts aim to provide more economically viable, efficacious, and secure therapeutic alternatives. In this review, we comprehensively scrutinize the clinical challenges associated with CAR-T cell therapy and provide an overview of potential strategies to overcome these obstacles. Our aim is to chart pathways for CAR-T cell therapy to navigate beyond its current limitations.

## 2 Achilles heels of CAR T-cell therapy

**Figure 1:**
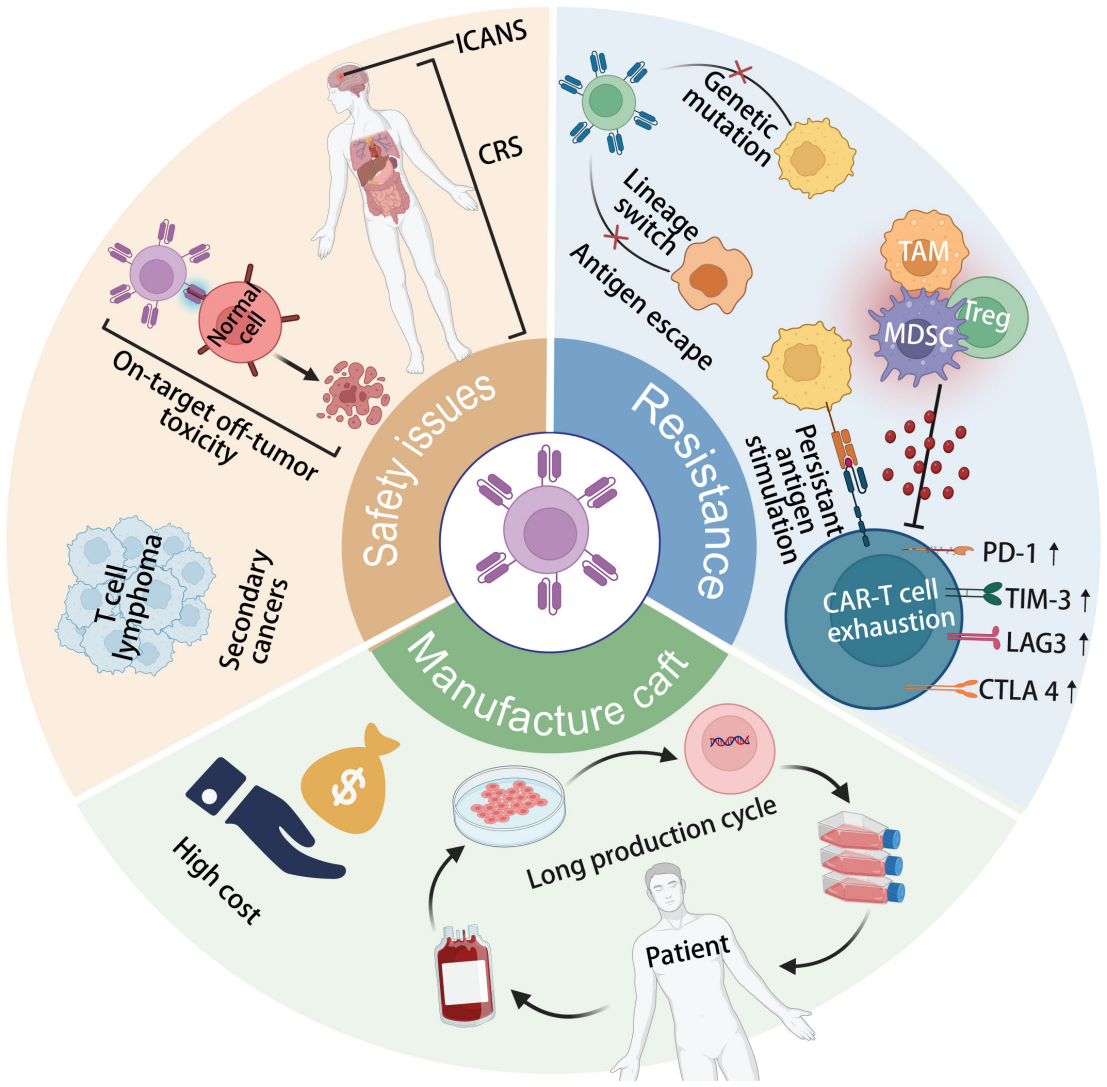
The current limitations of CAR-T cell therapy include tumor antigen escape, CAR-T cell exhaustion, secondary T-cell malignancies following treatment, cytokine release syndrome (CRS) and immune effector cell-associated neurotoxicity syndrome (ICANS) toxicity, on-target off-tumor toxicity, and complex, long-term manufacturing processes.

As depicted in **Figure 1**, the Achilles’ heels of CAR T-cell therapy, which currently hinder its wider efficacy and acceptance in clinical practice, encompass resistance to CAR-T cell therapy, safety concerns, and manufacturing intricacies (26, 37, 38). One major issue is tumor antigen escape, where cancer cells mutate or downregulate the target antigen recognized by CAR-T cells, leading to treatment resistance and disease relapse (37). Additionally, CAR-T cell exhaustion, characterized by decreased efficacy and persistence of infused cells over time, poses a significant challenge to long-term therapeutic success (39). On-target off-tumor toxicity is another concern, as CAR-T cells may inadvertently target healthy tissues expressing the target antigen, leading to adverse effects (29). Furthermore, the development of secondary T-cell malignancies following CAR-T cell treatment, though rare, underscores the need for continued vigilance regarding long-term safety outcomes (40). The toxicities associated with CRS and ICANS further complicate CAR-T therapy, requiring careful management to mitigate potentially life-threatening complications (41). Moreover, the complex and lengthy manufacturing processes involved in producing personalized CAR-T cell products limit their scalability and accessibility to patients (42). Addressing these Achilles’ heels is crucial for enhancing the overall efficacy, safety, and feasibility of CAR T-cell therapy in clinical settings.

### 2.1 Tumor antigen escape

While CAR-T cell therapy has demonstrated unprecedented response rates, not all patients benefit from it, and a significant percentage experience relapses (43). Antigen escape stands out as the most common cause of relapse in CD19-positive B-cell malignancies following CAR-T cell therapy. Published data indicate that CD19-negative relapse occurs in 7-25% of B-cell acute lymphoblastic leukemia (B-ALL) cases and approximately 30% of large B-cell lymphoma (LBCL) cases in patients treated with CD19 CAR therapy (4, 44–46). Mechanisms underlying CD19 loss have been extensively investigated, including CD19 gene mutations or splice variants, abnormal processing or trafficking of CD19 due to CD81 expression deficiency, and lineage marker switching from the lymphoid to the myeloid lineage (46, 47). Zah et al. developed a CD19-CD20 CAR and demonstrated its efficacy in preventing the spontaneous emergence of CD19-negative tumor cell variants in immune-deficient mice. In contrast to CD19, loss of B cell maturation antigen (BCMA) appears to be infrequent following anti-BCMA CAR-T cell therapy and has only been reported in a few studies (48). For instance, only 4% (3 out of 71) of patients in a phase II clinical trial using Idecabtagene Vicleucel for multiple myeloma (MM) treatment developed BCMA expression loss (12). Deletion and mutation of the biallelic TNFRSF17 gene encoding BCMA have been identified as the main mechanisms causing BCMA loss (49–51). To mitigate the risk of relapse and failed responses attributed to tumor antigen escape, it may be necessary to conduct more precise screening of the patient’s genetic spectrum before initiating a new CAR-T cell therapy (52).

### 2.2 CAR-T cell exhaustion

CAR-T cell exhaustion, a significant factor contributing to CAR-T cell resistance, often leads to antigen-positive relapse and results from various factors. Firstly, the differentiation status of T cells is crucial for maintaining the functional persistence of CAR-T cells. Previous studies have shown that less differentiated T cells have greater expansion potential and prolonged persistence compared to fully differentiated effector T cells (53). Most current CAR-T cell products are autologous, and due to factors, such as the presence of tumors, prior cytotoxic treatments, and prolonged ex vivo cultivation, these cells often exhibit an exhausted phenotype characterized by excessive differentiation (54, 55). Additionally, immunosuppressive components of the tumor immune microenvironment, including regulatory T cells, myeloid-derived suppressor cells, tumor-associated macrophages (TAMs), and immunosuppressive ligands, also contribute to CAR-T cell exhaustion (25, 56, 57). Furthermore, tonic CAR signaling transduction, mainly associated with the costimulatory domain, plays a key role in CAR-T cell exhaustion. Insufficient signaling compromises cell persistence, while excessive signaling leads to exhaustion (58). For example, compared to 4-1BB, CD28 CAR-T cells demonstrate faster and stronger cellular effector functions, but this rapid and intense signal transduction can induce CAR-T cell exhaustion, thereby limiting persistence (59–61). Achieving potent and sustained activity against specific tumor targets requires careful selection of optimal CAR designs. Recent studies have underscored the importance of positively charged plaques (PCPs) on CAR in mediating CAR aggregation at the antigen-binding domain surface, thereby facilitating sustained CAR signaling (62, 63). Regulating PCPs offers a means to optimize CAR-T cell function. For CARs with high sustained signaling, such as GD2.CAR and CSPG4.CAR, reducing PCPs during *in vitro* expansion or enhancing ionic strength in culture can diminish spontaneous CAR activation and mitigate CAR-T cell exhaustion. Conversely, for CARs with weak tonic signaling like CD19.CAR, augmenting PCPs on the CAR surface can enhance *in vivo* persistence and anti-tumor efficacy (62). Recently, the same team developed CAR-Toner, an artificial intelligence (AI)-based PCP score calculator and optimizer (64). Taking the camel single-domain nanobody (VHH) targeting the acute myeloid leukemia (AML) tumor-associated antigen CLL1 as an example, the authors optimized the CAR design using CAR-Toner to systematically reduce their PCP scores. The results showed that an intermediate tonic signaling strength optimally benefits CAR-T cell function. As an AI-based tool, CAR-Toner is capable of not only conducting PCP calculations but also offering optimization recommendations for PCP scores. This groundbreaking tool is anticipated to catalyze progress in the field of CAR-T design and significantly contribute to the advancement of AI-driven CAR-T design.

Various strategies have emerged to combat CAR-T cell exhaustion, such as blocking exhaustion-promoting signaling pathways, inhibiting downstream effectors, alleviating immunosuppression within the tumor microenvironment (TME), and converting inhibitory signals into stimulatory ones (52, 61). When coupled with AI-based tools, these approaches facilitate the development of more potent CAR designs, thus augmenting the efficacy of CAR-T cell therapy (65).

### 2.3 Secondary T-cell malignancies following treatment

Recent evidence indicates a concerning association between CAR-T cell therapy and the development of secondary T-cell malignancies, prompting regulatory measures by the FDA to enhance safety oversight (66, 67). By the close of 2023, 22 reported cases have documented the emergence of T-cell cancers subsequent to CAR-T treatment. Notably, in three instances, the CAR transgene was identified within the malignant clone, strongly implicating the therapy in the genesis of T-cell cancer (28). Presently, CAR-T products approved by regulatory agencies employ T cells engineered via viral transduction to convey the genetic construct. However, the use of current retroviral vectors still harbors potential risks of oncogenesis through genomic integration or related mechanisms. For example, lentiviral vector constructs, while integrating into the genome in a semi-random manner, display an affinity for genomic regions characterized by active gene expression, thereby heightening the risk of insertional oncogenesis (68). To mitigate these risks in the future, strategies such as precision targeting of CAR construct insertion to specific genomic loci or the utilization of transient CAR mRNA delivery to the cytoplasm offer promising avenues for enhancing safety in CAR-T therapy (68).

### 2.4 CRS and ICANS toxicity trigger by CAR-T cells

While CAR-T cell therapy holds tremendous promise in the therapeutic realm of hematologic malignancies, the associated potential life-threatening toxicities remain a significant concern. Cytokine release syndrome (CRS) and immune effector cell-associated neurotoxicity syndrome (ICANS) emerge as the two most common adverse events during CAR-T cell therapy (**Table 1**), attributed to the overactivation of CAR-T cells and the massive release of cytokines (69–71). CRS typically manifests a few days after the initial infusion, with symptoms ranging from mild flu-like manifestations such as fever, fatigue, chills, and muscle pain to severe life-threatening complications including shock, hypotension, coagulation abnormalities, and multi-organ dysfunction (72, 73). On the other hand, ICANS typically occurs 1-3 weeks post-CAR-T cell infusion, characterized by symptoms such as aphasia, delirium, focal neurological deficits, tremors, seizures, or life-threatening cerebral edema (74, 75). The severity of CRS and ICANS is categorized from grade I to grade IV, depending on factors such as CAR structure, CAR-T cell dosage, tumor burden, treatment targets, and patients’ individual characteristics. Most patients receiving CAR-T cell therapy are likely to experience varying degrees of CRS, with nearly half developing ICANS (**Table 1**). Among these patients, 10%-40% may experience severe toxicity reactions (≥ grade 3), necessitating admission to the intensive care unit (ICU) for life support and the use of additional medications such as the interleukin (IL)-6 receptor antagonist tocilizumab (76–78). Blocking cytokine networks or optimizing the structural design of CARs to reduce toxicity are potential strategies to mitigate CRS and ICANS triggered by CAR-T cells (79, 80).

### 2.5 On-target off-tumor toxicity

Given that most CAR-T cell target antigens are not exclusively tumor-specific and are also expressed in normal cells, CAR-T cells may inadvertently cause damage to normal tissue and organs while targeting tumors (81). Long-term follow-up data indicate that on-target off-tumor toxicity, leading to B-cell aplasia and hypogammaglobulinemia, are the most common long-term adverse reactions following treatment with anti-CD19 CAR-T cells (14). Although these side effects can be managed through sequential intravenous immunoglobulin, the long-term repeated infusions may escalate treatment costs. Additionally, cytopenia, infections, and tumor lysis syndrome are also common side effects of CAR-T cell therapy for hematologic malignancies. While proper management and intervention can control these toxicities, the resulting disease and economic burden are significant factors limiting the widespread adoption of CAR-T cell therapy (77, 82). Additionally, multiple-targeting CAR-T cells are designed to mitigate the impact of on-target off-tumor effects. Intensive research has centered on developing diverse protein-based logic-circuit strategies to enhance the specificity of CAR T cell activation and cytotoxicity towards tumor cells (83–85). Additionally, locoregional administration of CAR T cells, aimed at concentrating antitumor activity within the tumor microenvironment, may offer a potential solution to mitigate off-tumor toxicity (86–88).

### 2.6 Complexed and long-term manufacturing process

**Figure 2:**
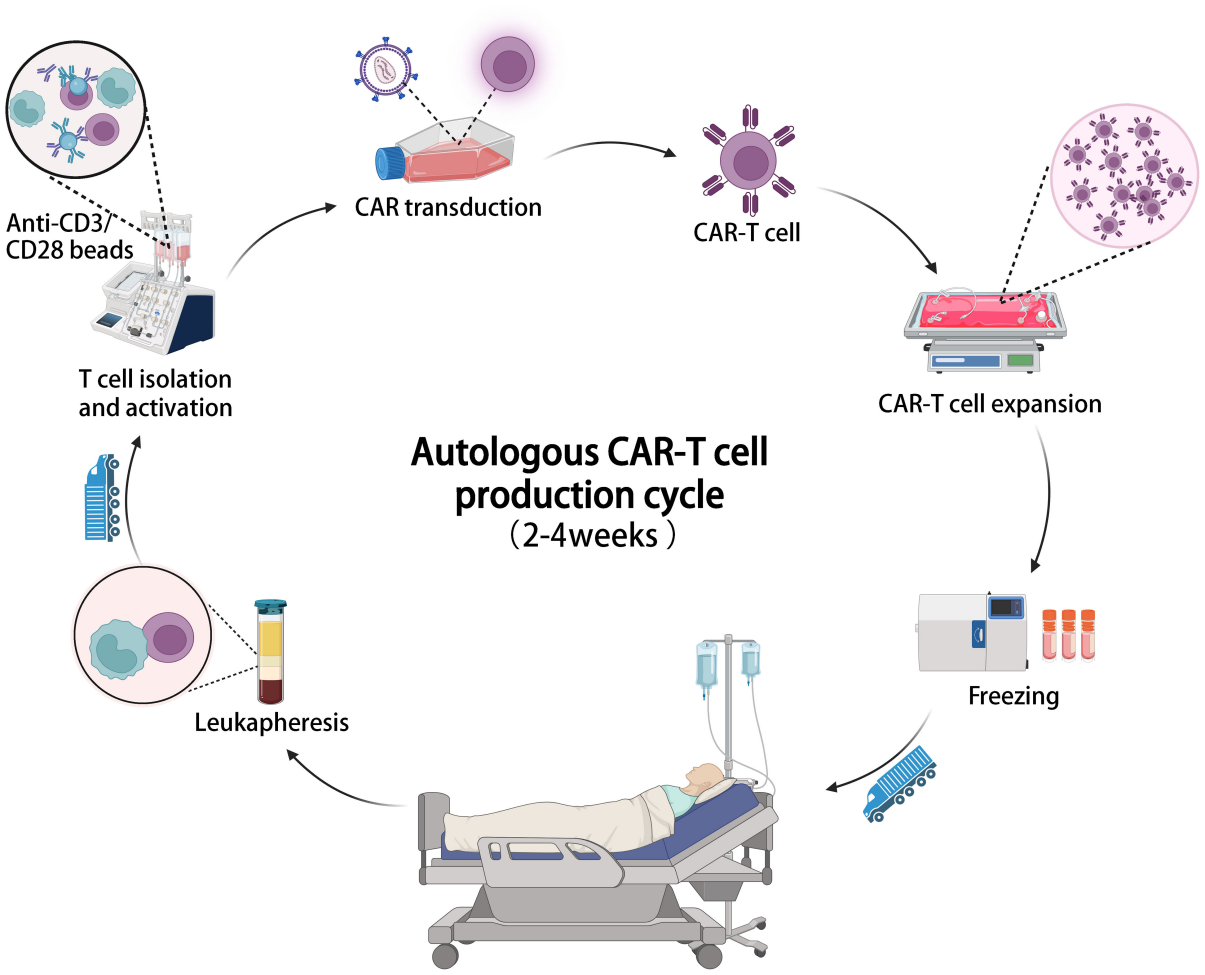
The illustration of the entire manufacturing process of autologous CAR-T cell therapy, which typically takes 2-4 weeks from cell collection to infusion.

A major challenge of CAR-T cell therapy is its high cost and lengthy manufacturing process. Most FDA-approved and clinical trial CAR-T cell products are autologous, requiring personalized customization involving complex processes like T cell isolation, activation, CAR gene transduction, expansion, and reinfusion (89). This labor-intensive process occurs in specialized facilities, adding to expenses and time. Viral vector preparation, crucial for CAR transfection, also contributes to costs and production delays (90). Production of viral vectors requires at least 2 weeks, meeting cGMP standards and extensive safety testing (91–93). The cost of a single dose of approved CAR-T cell products ranges from $373,000 to $475,000 (**Table 1**), with a production cycle of 2 to 4 weeks (**Figure 2**), posing economic burdens and logistical challenges for patients (94). With advancements in related technologies, it is imperative to establish increasingly standardized and simplified manufacturing processes to mitigate the costs associated with CAR-T therapy (22, 95).

## 3 New paradigms for CAR-T cell therapy beyond current Achilles heels

### 3.1 Allogeneic universal CAR-T cells

**Figure 3:**
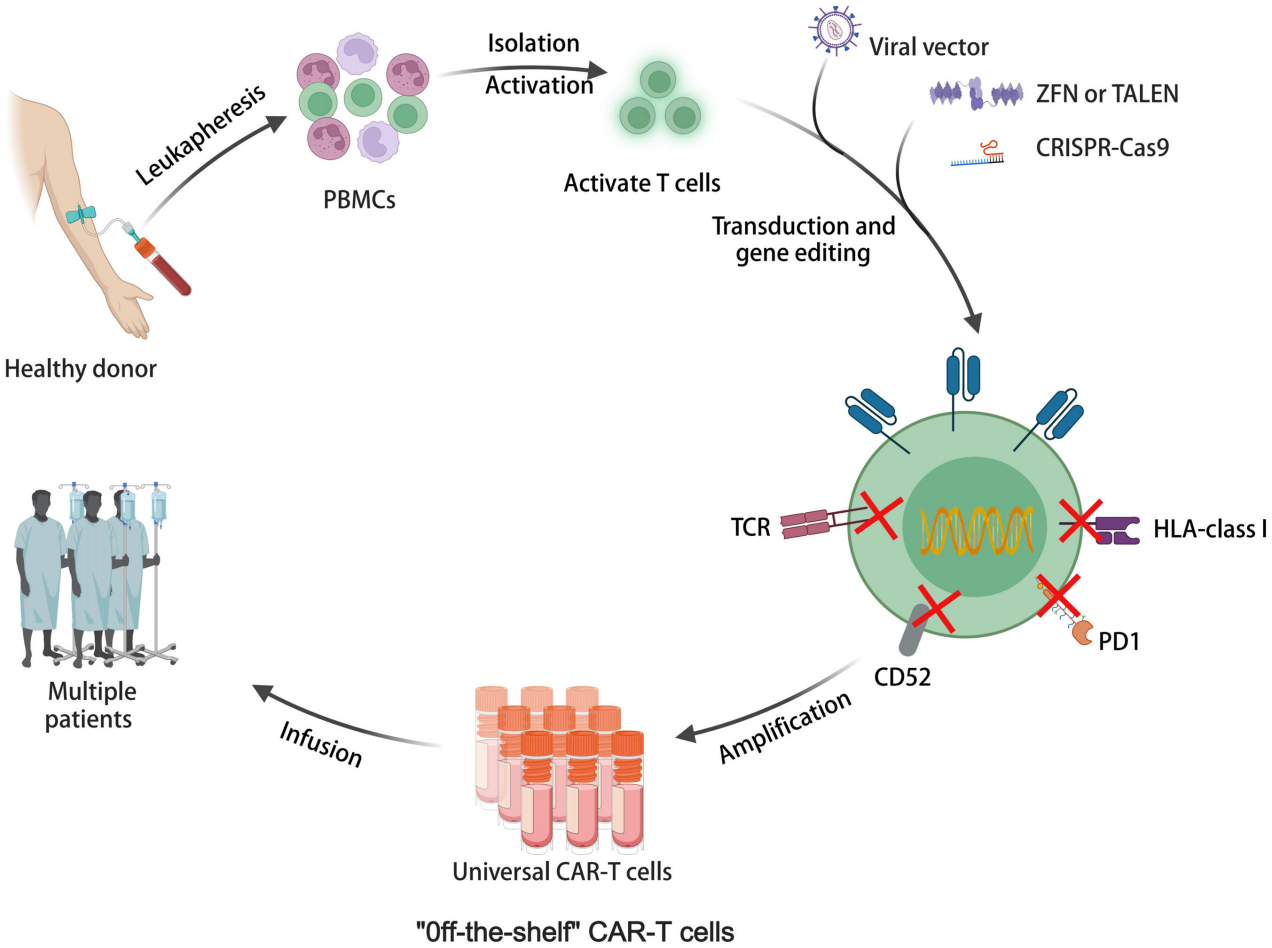
The illustration of allogeneic universal CAR-T cell therapy for off-the-shelf cell production.

Allogeneic CAR-T cells sourced from healthy donors offer advantages such as scalable production, lower manufacturing costs, and immediate availability (96). As illustrated in **Figure 3**, allogeneic universal CAR-T cells represent off-the-shelf cell production. They are undergoing testing in numerous clinical trials, with promising outcomes documented in **Table 2**. For instance, a meta-analysis showed CR rates of 70% in R/R ALL and 52% in non-Hodgkin lymphoma (NHL) with CD19 CAR-T cells derived from healthy donors (97). However, allogeneic CAR-T cells face challenges like T-cell receptor (TCR)-mediated graft-versus-host disease (GVHD) and human leukocyte antigen (HLA) related host-versus-graft (HvG) response due to their allogeneic nature (96). Targeted knockout of TCR, HLA, and related molecules using gene editing techniques is an effective strategy. Various gene editing tools have been explored, including zinc finger nucleases (ZFNs) (98), transcription activator-like effector nucleases (TALENs) (99), and CRISPR-Cas9 (100). CRISPR-Cas9 stands out for its versatility, allowing for simultaneous editing of multiple genes. It has been extensively used in allogeneic CAR-T cells, targeting TCR and HLA class I molecules, and guiding CAR insertion to specific loci, enhancing CAR-T cell potency and stability (101). However, CRISPR-Cas9 editing has limitations such as off-target editing and large-scale genomic rearrangements. Base editing technology, a precise gene editing tool based on CRISPR, offers new possibilities for overcoming these challenges without inducing double-stranded DNA breaks (DSBs) (102, 103). Adenine base editors (ABEs) and cytosine base editors (CBEs) enable the conversion of specific DNA bases without DSBs (104). Recent studies have shown the feasibility of highly specific knockout of T cell genes using base editing, paving the way for the development of allogeneic CAR-T cells with enhanced safety and efficacy (105). While allogeneic CAR-T cell therapy holds promise, ongoing clinical trials are essential to further evaluate its safety and efficacy. Longer-term follow-up data from these trials will provide valuable insights into the durability of responses and the potential for adverse events. Continued research efforts are necessary to optimize allogeneic CAR-T cell therapies and address any challenges that may arise, ultimately advancing their clinical utility in treating various malignancies (106).

**Table 2:** Currently registered clinical trials of genome-edited allogeneic CAR-cell products.

Gene editing tools	Target Antigen	Product	Knockout loci	Indication	Clinical trial phase	Clinical trial number	Study Start
TALEN	CD19	UCART19	TRAC and CD52	R/R B-ALL R/R B-ALL R/R BCL R/R LBCL/FL	Phase 1 Phase 1 Phase 1/2 Phase 1	NCT02808442 NCT02746952 NCT03166878 NCT03939026	Jun 3, 2016 Aug 1, 2016 Jun, 23, 2017 May 1, 2019
	CD19	ALLO-501A	TRAC and CD52	R/R LBCL/CLL/SLL	Phase 1/2	NCT04416984	May 21, 2020
	BCMA	ALLO-605	TRAC and CD52	R/R MM	Phase 1/2	NCT05000450	Jun 6, 2021
	BCMA	ALLO-715	TRAC and CD52	R/R MM	Phase 1	NCT04093596	Sep 23, 2019
	CD123	UCART123	TRAC	R/R AML R/R BPDCN	Phase 1 Phase 1	NCT03190278 NCT03203369	Jul 19, 2017 Jul 28, 2017
	CD22	UCART22	TRAC and CD52	R/R B-ALL	Phase 1	NCT04150497	Oct 14, 2019
	CS1	UCARTCS1	TRAC and CS1	R/R MM	Phase 1	NCT04142619	Nov 21, 2019
CRISPR/Cas9	CD7	WU CART-007	TRAC and CD7 TRAC and CD7	T-ALL/LBCL T-NHL/AML/TCL/ATL	Phase 1/2 Phase 1	NCT04984356 NCT05377827	Jan 14, 2022 Oct 10, 2023
	CLL1	CB-012	TRAC, β2M, PDCD1	R/R AML	Phase 1	NCT06128044	Dec 20, 2023
	CD19	UCART019	TRAC and β2M	R/R BCL	Phase 1/2	NCT03166878	Jun, 2017
	CD19	PACE CART19	TRAC, B2M and CIITA	R/R BCL	Phase 1	NCT05037669	Jul, 2022
	CD19	PBLTT52CAR19	TRAC and CD52	R/R BLL	Phase 1	NCT0455743	Aug 12, 2020
	CD19	SC291	TRAC, B2M and CIITA	R/R NHL/CLL	Phase 1	NCT05878184	Apr, 2024
	CD19	CTA101	TRAC and CD52	R/R B-ALL R/R B-ALL/NHL	Phase 1 Early Phase 1	NCT04154709 NCT04227015	Dec 10, 2019 Jan 8, 2020
	CD19	ATHENA	TRAC and Power3	R/R NHL	Phase 1/2	NCT06014073	Sep 6, 2023
	CD19	CTX110	TRAC and β2M	R/R BCL/NHL	Phase 1/2	NCT04035434	Jul 22, 2019
	CD19	CB-010	TRAC, PD-1	R/R BCL	Phase 1	NCT04637763	May 26, 2021
	BCMA	CB-011	TRAC and β2M	R/R MM	Phase 1	NCT05722418	Feb 6, 2023
	BCMA	CTX120	TRAC and β2M	R/R MM	Phase 1	NCT04244656	Jan 1, 2020
	CD70	CTX130	TRAC and β2M	R/R TCL	Phase 1	NCT04502446	Jul 31, 2020
	CD19/CD7	GC502	TRAC and CD7	R/R BCL	Early Phase 1	NCT05105867	Sep 29, 2021
CRISPR-CLOVE	BCMA	P-BCMA-ALLO1	TCR and β2M	R/R MM	Phase 1	NCT04960579	May 5, 2022
Base editing	CD7	BEAM-201	CD7, TRAC, PDCD1 and CD52	R/R T-ALL/T-LL	Phase 1/2	NCT05885464	May 25, 2023
	CD7	BE-CAR7	CD7, TRAC, and CD52	R/R T-ALL	Phase 1	ISRCTN15323014	Sep 7, 2023
ARCUS	CD19	PBCAR19B	TCR	R/R BCL	Phase 1	NCT04649112	Jun 16, 2021
	CD19	PBCAR0191	TCR	R/R NHL/ALL	Phase 1/2	NCT03666000	Mar 11, 2019
	BCMA	PBCAR269A	TCR	R/R MM	Phase 1	NCT04171843	Apr 30, 2020
	CD20	PBCAR20A	TCR	R/R NHL/CLL/SLL	Phase 1/2	NCT04030195	Mar 24, 2020

R/R, refractory or relapsed; B-ALL, B-cell acute lymphoblastic leukemia; LBCL, large B Cell Lymphoma; FL, follicular lymphoma; CLL, chronic lymphocytic leukemia; SLL, small lymphocytic lymphoma; MM, multiple myeloma; AML, acute myeloid leukemia; BPDCN, Blastic Plasmacytoid Dendritic Cell Neoplasm; NHL, non-Hodgkin lymphoma; TCL, T-Cell Lymphoma; ATL, adult T-cell leukemia; BCL, B Cell Lymphoma/Lymphoma; -NHL, T-Cell non-Hodgkin lymphoma; T-ALL, T-cell acute lymphoblastic leukemia; T-LL, T-Cell Lymphoblastic Lymphoma.

### 3.2 Rapid manufacturing of autologous CAR-T cells

While significant progress has been made in the exploration of allogeneic and *in vivo* CAR-T cells, they remain in continuous proof-of-concept stages without clinical approval. Therefore, enhancing product quality, refining manufacturing processes, and reducing self-production time are crucial to address the challenges faced by autologous CAR-T cells. Shortening the ex vivo culture time has been shown to yield CAR-T cells with improved effector function and reduced production costs (54). Several promising methods for rapid CAR-T cell production have been explored, such as the FasTCAR platform by Gracell Biotechnologies, which produces CAR-T cells within a day and has demonstrated efficacy in preclinical and clinical evaluations for R/R B-ALL (35). Additionally, researchers at the University of Pennsylvania have successfully prepared functional CAR-T cells within 24 hours by directly transducing non-activated quiescent T cells (by lentivirus vector), extending the survival of tumor-bearing mice (107). Agarwalla et al. have developed an implantable Multifunctional Alginate Scaffold for T Cell Engineering and Release (MASTER), which integrates T cell activation, reprogramming, and *in vivo* expansion, reducing manufacturing time to 1 day. CAR-T cells produced using the MASTER scaffold have shown promising anti-tumor activity in mouse xenograft models of lymphoma (36). These rapidly manufactured CAR-T cells exhibit superior anti-tumor activity and greater persistence compared to conventional CAR-T cells, potentially offering a more cost-effective approach.

### 3.3 *In vivo* induced CAR-T cell therapy

**Figure 4:**
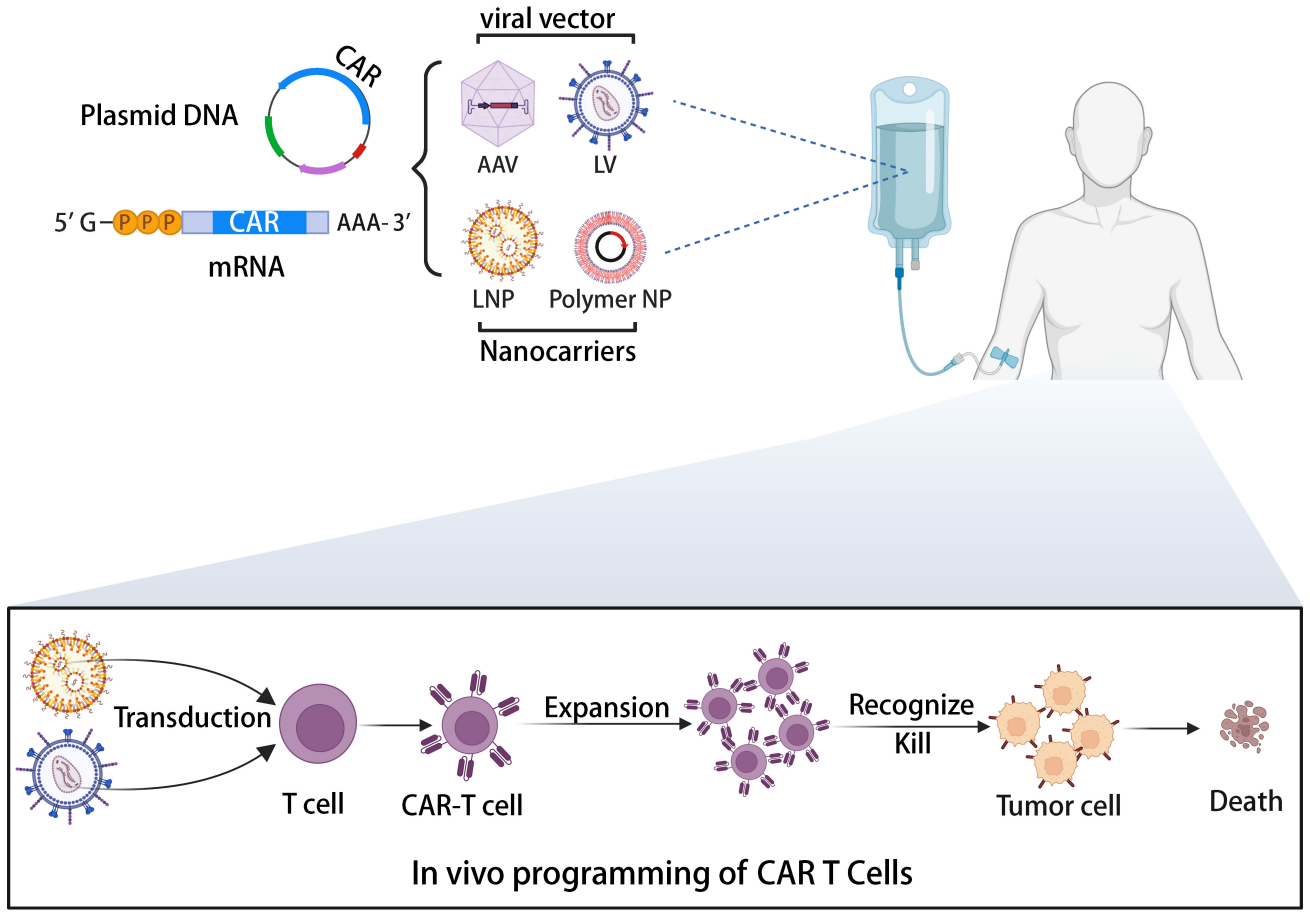
The illustration of the *in vivo* induction of CAR-T cells utilizing viral vectors or non-viral nanocarriers.

Allogeneic universal CAR-T cells, while offering solutions to immune rejection through gene editing, also raise safety concerns. A previous clinical trial of allogeneic CAR-T therapy was halted by the FDA due to the emergence of chromosomal abnormalities (108). Although investigations suggested the abnormality wasn’t related to gene editing, safety concerns persist. A potential solution lies in direct CAR-T cell generation in patients via a universally applicable medicinal product containing the CAR gene. The illustration of *in vivo* generation of CAR-T cells is depicted in **Figure 4** (109). This approach’s combination of simplicity, speed, and cost-effectiveness makes it an attractive option for CAR-T cell therapy. By leveraging the body’s natural processes, *in vivo* CAR-T cell generation eliminates the requirement for ex vivo cell manipulation and lengthy manufacturing processes. This streamlined method not only reduces production time but also lowers associated costs, potentially improving the accessibility of CAR-T cell therapy to a broader patient population. Various vector platforms are under exploration, including lentiviral vectors (LVs) and nanoparticles (NPs) (110, 111). Lentiviral vectors (LVs) stand as the predominant choice for ex vivo CAR-T cell transduction, boasting stable gene integration, high transduction efficiency, and a broad host range. Pioneering work by Buchholz et al. introduced the pseudotyping of vectors with modified envelope proteins of Nipah virus (Niv), enabling specific targeting of CD8 by fusing the envelope protein to a CD8-specific single-chain variable fragment (scFv) (112). Their study demonstrated that CD8-LV could directly generate human CD19-CAR-T cells in NGS mice *in vivo*, showcasing potent anti-tumor activity. Furthermore, Buchholz et al. developed Niv-LV targeting CD3 and CD4, capable of directly generating functional CAR-T cells in mice (113). Despite LVs’ high transduction efficiency, the potential risk of insertional mutagenesis remains a concern (114). In recent years, researchers have explored virus-like particles (VLPs) as a novel vector. VLPs retain viral proteins without containing a packaged genome, combining viral vector targeting specificity with the transient delivery advantages of non-viral vectors. Hamilton et al. demonstrated the generation of gene-edited CAR-T cells *in vivo* by packaging Cas9 RNPs into retroviral VLPs, offering a new direction for future *in vivo* CAR-T cell generation, albeit with potentially lower *in vivo* transduction efficiency compared to LVs (115). Nanoparticles (NPs) have garnered significant attention as gene delivery vehicles due to their low immunogenicity, cost-effectiveness, and customizable production (116). Unlike the complex machinery of viruses, gene delivery using NPs relies on the physicochemical properties of the particles and payloads, offering advantages such as payload flexibility and ease of modification (117). The two primary types of nanocarriers used in CAR-T cell development are lipid- and polymer-based NPs. Numerous preclinical studies have successfully employed these carriers to deliver CAR gene-containing DNA or mRNA into T cells *in vivo*, leading to the on-site generation of CAR-T cells and effective cytotoxic functions in small animal models. For instance, Matthias T. Stephan’s team designed polymer nanoparticles encapsulating CAR DNA and mRNA (118, 119), resulting in anti-tumor efficacy comparable to CAR-T cells prepared using traditional lentiviral vectors. In addition to antibody-targeted nanocarriers, Daniel J. Siegwart’s team recently introduced a Selective Organ Targeting (SORT) LNP capable of delivering mRNA to the spleen and locally generating CAR-T cells in a controlled manner (120). The research into *in vivo* generation of CAR-T cells has the potential to transform CAR-T cell therapy into a widely adopted pharmaceutical treatment (109). This advancement could significantly enhance clinical compliance and substantially reduce costs (121). Amid the FDA’s recent warning regarding secondary cancer risks following CAR-T cell infusion treatments, the application of *in vivo* editing to transiently modify CAR-T cells using mRNA offers a promising, potentially safer, and cost-effective solution to address the existing challenges associated with CAR-T cell therapy (122). By harnessing AI-based tools to refine CAR design, the advancement of *in vivo* CAR-T cell development stands poised to accelerate, offering a more streamlined and effective approach toward overcoming the current challenges in CAR-T cell therapy (123, 124).

## 4 Conclusions and prospects

CAR-T therapies have demonstrated unprecedented efficacy in the treatment of hematologic malignancies. However, their widespread application is impeded by high costs, lengthy preparation time, safety concerns, and limited effectiveness. To overcome these obstacles, a revolution in gene editing tools and delivery vectors is necessary to establish new paradigms for CAR-T cell therapy that surpass current Achilles heels. With the ongoing refinement of gene editing tools and delivery vectors, highly potent and super safe CAR-T cells are poised to become widely utilized in clinical settings akin to conventional living drugs in the future. The expansion and exploration of these technologies are opening new possibilities for CAR-T cell therapy, offering patients more efficient, secure, and affordable therapeutic options.

## Author contributions

YL: Investigation, Software, Writing – original draft. ZH: Conceptualization, Methodology, Writing – original draft, Writing – review & editing. YYL: Funding acquisition, Methodology, Supervision, Writing – review & editing. XW: Conceptualization, Funding acquisition, Resources, Writing – review & editing.
